# Intrauterine Growth Restriction: New Insight from the Metabolomic Approach

**DOI:** 10.3390/metabo9110267

**Published:** 2019-11-06

**Authors:** Elena Priante, Giovanna Verlato, Giuseppe Giordano, Matteo Stocchero, Silvia Visentin, Veronica Mardegan, Eugenio Baraldi

**Affiliations:** 1 Neonatal Intensive Care Unit, Department of Women’s and Children’s Health, University of Padua, 35128 Padua, Italy; giovanna.verlato@aopd.veneto.it (G.V.); veronica.mardegan@aopd.veneto.it (V.M.); eugenio.baraldi@unipd.it (E.B.); 2 Department of Women’s and Children’s Health, University of Padua, 35128 Padua, Italy; giuseppe.giordano@unipd.it (G.G.); matteo.stocchero@unipd.it (M.S.); 3 Institute of Pediatric Research, “Città della Speranza” Foundation, 35129 Padua, Italy; 4 Gynecology and Obstetrics Unit, Department of Women’s and Children’s Health, University of Padua, 35128 Padua, Italy; visentin78@gmail.com

**Keywords:** intrauterine growth restriction, fetal growth restriction, small for gestational age, metabolomics, newborn, nuclear magnetic resonance spectroscopy, mass spectrometry, biomarkers

## Abstract

Recognizing intrauterine growth restriction (IUGR) is a matter of great concern because this condition can significantly affect the newborn’s short- and long-term health. Ever since the first suggestion of the “thrifty phenotype hypothesis” in the last decade of the 20th century, a number of studies have confirmed the association between low birth weight and cardiometabolic syndrome later in life. During intrauterine life, the growth-restricted fetus makes a number of hemodynamic, metabolic, and hormonal adjustments to cope with the adverse uterine environment, and these changes may become permanent and irreversible. Despite advances in our knowledge of IUGR newborns, biomarkers capable of identifying this condition early on, and stratifying its severity both pre- and postnatally, are still lacking. We are also still unsure about these babies’ trajectory of postnatal growth and their specific nutritional requirements with a view to preventing, or at least limiting, long-term complications. In this setting, untargeted metabolomics—a relatively new field of ‘-omics’ research—can be a good way to investigate the metabolic perturbations typically associated with IUGR. The aim of this narrative review is to provide a general overview of the pathophysiological and clinical aspects of IUGR, focusing on evidence emerging from metabolomic studies. Though still only preliminary, the reports emerging so far suggest an “early” pattern of glucose intolerance, insulin resistance, catabolite accumulation, and altered amino acid metabolism in IUGR neonates. Further, larger studies are needed to confirm these results and judge their applicability to clinical practice.

## 1. Introduction

The first thousand days of life—between conception and a child’s second birthday—are a delicate time, when the health foundations of a lifetime are laid. Appropriate nourishment during this crucial period of life has a profound impact not only on the early life growth and development, but also on the lifelong health of the individual [1]. This concept should be expanded to include more than just nutrition in the strict sense, as the whole environment to which the infant is exposed is extremely important. Intrauterine growth restriction (IUGR) is a paradigmatic condition in which a “hostile intrauterine environment” can hamper fetal development with a potential impact on long-term health [2].

The emerging term “exposome” refers to the whole set of nongenetic exposures that, together with the genome, determine the final phenotype during the course of a lifetime [3]. Untargeted metabolomics is evolving as a novel tool for studying the effects of the endogenous and exogenous environment. Taking an untargeted hypothesis-generated approach, metabolomics enables the simultaneous qualitative and quantitative analysis of thousands of different metabolites in a biological sample, enabling the identification of biomarkers and metabolic patterns characteristic of a given condition. Among the “-omic” sciences, metabolomics is the one that comes closest to phenotyping. Such studies are typically performed on biofluids, which are analyzed with platforms such as nuclear magnetic resonance (NMR) spectroscopy or mass spectrometry (MS). Unexpected, or even unknown, metabolites may be revealed as important in characterizing specific groups of individuals, prompting new pathophysiological hypotheses on the condition under study [4].

In this narrative review, we first present some key aspects of the pathophysiology and clinical presentation of IUGR. Then, we focus on all the evidence emerging from metabolomic studies published so far. We take a critical look at the statistical and bioinformatic tools used to analyze the metabolomic data in the studies forming the object of our review. The aim of this approach is to give readers an adequate overview on IUGR, since we strongly believe that understanding the complex data deriving from metabolomic analyses cannot be separated from a thorough knowledge of the pathophysiology of the disease being considered.

### 1.1. IUGR: Definition and Obstetric Diagnosis

IUGR is estimated to affect between 5% and 10% of all pregnancies worldwide [2], and it is a condition that can strongly influence the newborn’s short- and long-term health.

When dealing with IUGR, the first difficulty is posed by its definition. IUGR refers to a condition in which a fetus is unable to achieve its genetically determined potential size. Although the term is often used interchangeably with the condition of Small for Gestational Age (SGA), IUGR is a prenatal finding of growth restriction, confirmed by a documented low fetal growth rate and/or by the presence of specific causes such as fetal infection, genetic abnormalities, an impaired placental blood flow, or toxicities. IUGR may be asymmetrical or symmetrical in nature, depending on the timing of the prenatal insult and diagnosis [5]. An international consensus effort has recently defined fetal growth restriction (FGR) as a fetus positive for at least one of the following parameters at <32 weeks: (i) an abdominal circumference <3rd centile; (ii) an estimated fetal weight <3rd centile; (iii) no end-diastolic flow in the umbilical artery; (iv) an abdominal circumference/estimated fetal weight ratio <10th centile combined with a pulsatility index (PI) >95th centile in the umbilical and/or uterine artery) (see reference for definition of FGR in a fetus at >32 weeks). Fetuses with congenital anomalies are excluded from this classification [6]. On the other hand, SGA refers to a postnatal condition when a baby’s birth weight is below the 10th centile for gestational age. Both these definitions have their weaknesses. Considering birth weight alone can lead to an overestimation of cases of SGA, with the inclusion of newborns who are simply constitutionally small but do not risk adverse outcomes. On the other hand, considering only prenatal assessments can result in some cases going undiagnosed due to a growth restriction or arrest occurring shortly before delivery that may not be detected by ultrasound [7]. Ideally, our attention should shift from single growth parameters (head circumference, abdominal circumference, weight, etc.) to assessing a fetus’ growth potential. In fact, individualized growth models have now been developed to predict third-trimester growth trajectories on the strength of second-trimester growth rate measurements [8]. It is noteworthy that 42% of 126 singletons judged to be SGA using conventional methods were actually found to be growing normally when assessed with individualized growth models [9].

### 1.2. Short- and Long-Term Outcomes for IUGR Neonates

**Figure 1:**
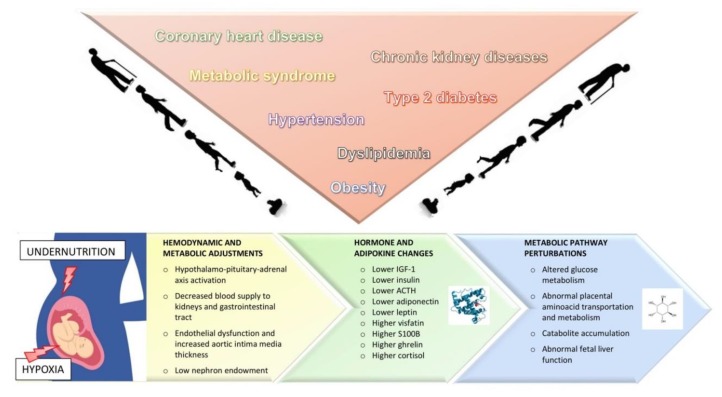
Summary of current knowledge on the short- and long-term consequences of intrauterine growth restriction. The fetus’ prenatal exposure to undernutrition and hypoxia induce hemodynamic and metabolic adjustments, as well as changes in hormones and adipokines. IGF-1: Insuline-like growth factor; ACTH: adrenocorticotropic hormone.

For SGA infants, the mortality risk is 2 to 4 times higher than for infants born at term or preterm but not SGA [10]. For growth-restricted infants, the immediate consequences at delivery may include hypothermia, hypoglycemia, hyperglycemia, persistent pulmonary hypertension, pulmonary hemorrhage, polycythemia, stillbirth, and intrapartum asphyxia [11]. Since IUGR is frequently associated with preterm birth, multisystem diseases of prematurity, such as impaired physical growth, or cognitive and motor development, may affect the intermediate and long-term outcomes of IUGR newborns. Several studies also suggest that an impaired fetal growth can predispose to certain major diseases later in life, including metabolic syndrome, obesity, coronary heart disease, hypertension, dyslipidemia, type 2 diabetes, and chronic lung and kidney diseases [12,13,14,15] (Figure 1). The assumption is that the fetus makes a number of hemodynamic and metabolic adjustments to cope with the adverse uterine environment, which may lead to permanent changes in the function and structure of several organ systems. Postnatally, when the environmental conditions change, the growth-restricted newborn’s response might be inadequate, further raising the risk of long-term consequences [16].

A few decades ago, Hales and Barker advanced the hypothesis of the so-called “thrifty phenotype”, according to which insulin resistance and type 2 diabetes during adulthood might originate from a fetal adaptation to save glucose in response to intrauterine malnutrition [17]. Chronic diseases in adulthood may actually be the consequence of an “altered programming”, when a stimulus or insult at a critical time in early life has permanent effects on structure, physiology, and metabolism. Previous studies have shown that particular body proportions at birth can provide a stronger and more specific prediction of later disease than birth weight alone [18]. IUGR fetuses have a disproportionately low fat mass compared to their lean mass [19], and an impaired skeletal muscle development [20]. When Barker studied various birth measurements, abdominal circumference was the one best able to predict plasma concentrations of total and LDL cholesterol, and apolipoprotein B. A small abdominal circumference at birth reflects liver size and, since cholesterol metabolism is regulated by the liver, we can infer that an impaired liver growth in utero realigns cholesterol concentrations with a more atherogenic profile [21].

Prenatal exposure to undernutrition and hypoxia prompts activation of the hypothalamo–pituitary–adrenal axis as an adaptive response to stress. Analyses on cord blood samples have revealed higher cortisol and lower adrenocorticotropic hormone (ACTH) levels in cases of IUGR [22]. Fetal growth restriction leads to a redistribution of the blood flow by reducing the supply to the kidneys and gastrointestinal tract and preserving the flow to the brain, myocardium, and adrenal glands [16]. Low birth weight has also been associated with endothelial dysfunction [23] and an increased aortic intima media thickness has been found in IUGR neonates on ultrasound [24].

An enhanced insulin sensitivity at birth, followed by an accelerated postnatal growth and the subsequent emergence of insulin resistance, is another feature of IUGR neonates [25]. Cord blood insuline-like growth factor (IGF-1) and insulin levels were found lower for IUGR newborns than for babies whose size was Adequate for Gestational Age (AGA) [26].

Interestingly, fetal macrosomia coincides with similar alterations in terms of fetal adipose tissue development, permanent changes in hormone function regulation, and a strong tendency for obesity and related metabolic disorders later in life [27].

In addition to IUGR newborns’ birth weight, their weight gain during infancy and early childhood was recently found to be related to their body composition and cardiovascular risk in adolescence [28]. A higher cholesterol synthesis at 8–12 years of age was reported in infants with a greater catch-up growth [29]. Higher blood pressure at 6–8 years old was reported in a group of term-born infants judged to be SGA and given a nutrient-enriched formula (containing 28% more protein) [30]. While an excessive calorie intake and weight gain early in life can negatively affect long-term health, postnatal nutrient restriction may lead to complications as well. Despite considerable efforts and advances in the management of nutritional aspects in newborns, it is not uncommon to find evidence of malnutrition, extrauterine growth restriction [31], and bone disease [32] in Neonatal Intensive Care Units. Premature babies in general, and IUGR newborns in particular, are often discharged from hospital with a body weight and length below the norm for gestational age [33]. This deficit may restrict the huge increase in fetal body weight and brain volume normally occurring at this time of life [34]. In-hospital growth correlates positively with neurological outcome [35,36], and energy and protein intakes in the first week of life are each independently associated with a newborn’s Mental Development Index (MDI). Malnutrition during rapid brain growth results in fewer neurons, which might lead to future behavioral problems and memory and learning difficulties [37,38]. Improving nutrition can increase the likelihood of survival without neurodevelopmental impairment in Very Low Birth Weight infants [39]. Judging from these findings, it seems that both overnutrition and undernutrition at a crucial time of life are associated with adult diseases. It is mandatory to promote adequate growth after birth, but we still do not know how to judge the quality of an infant’s growth—especially in the case of newborns who have suffered from IUGR. We also lack reliable biomarkers that can tell us whether the nutritional status and growth rate of a preterm baby are appropriate.

### 1.3. Pre- and Postnatal Biomarkers of Intrauterine Growth Restriction

Many efforts have been made to address the early detection or prediction of fetal growth restriction in the early stages of pregnancy with a view to implementing prophylactic strategies and stricter monitoring. β-human chorionic gonadotropin (β-HCG), pregnancy-associated plasma protein-A (PAPP-A), placental growth factor (PlGF), and soluble fms-like tyrosine kinase-1 (sFlt-1) are some of the biochemical analytes in the serum studied so far as potential predictors of placental dysfunction in early pregnancy. Unfortunately, when used alone, the value of these biomarkers in predicting IUGR is rather low. Combining these maternal serum markers with abnormal Doppler findings has been shown to increase their accuracy and sensitivity in predicting adverse outcomes—especially for IUGR and pre-eclampsia—but their use has yet to take hold in clinical practice [40,41]. The search for biomarkers of IUGR has also focused on the newborn immediately after birth. Dessì et al. summarized some of the mediators found to be related to newborn anthropometry at birth [42] (Figure 1). Levels of leptin and adiponectin (two proteins secreted mostly by fat tissue) also correlate with birth weight [43]. In addition, adiponectin levels have been found significantly higher in AGA than in SGA neonates [44]. A recent study confirmed a positive association between leptin (but not adiponectin) levels and anthropometric parameters in IUGR twins born from discordant dichorionic pregnancies [45]. One interesting finding concerns the higher concentrations of visfatin (a protein produced by visceral fat and associated with insulin resistance) in IUGR neonates than in those with a normal birth weight: this is probably due to a greater visceral adiposity or an altered fetal development of adiposity in cases of IUGR [46,47]. Florio et al. found increased urinary levels of S100B (an inflammatory protein expressed in cerebral and adipose tissue) in IUGR newborns [48]. Levels of S100B and neuron-specific enolase (another known brain injury biomarker) seem to relate to worse neurodevelopmental scores at two years of age, suggesting a possible link between these molecules and the neurological sequelae observed in IUGR [49]. Ghrelin, a hormone with orectic properties, has been found in higher-than-normal levels in cord blood from SGA babies: this is likely to be an adaptive response to malnutrition in fetal life [50]. Epigenetic changes have also been demonstrated: IUGR neonates show significant DNA hypomethylation when compared with AGA newborns [51].

## 2. Methods

The main research question for this review was: “Is metabolomics a good tool for identifying new markers of IUGR?” We searched (2000–2019) Pubmed, Web of Science, and Google Scholar for publications on metabolomics applied to IUGR research. “Intrauterine growth restriction”, “fetal growth restriction”, “newborn”, and “metabolomics” were used as key words. We also expanded our search by following up the references in the articles identified. Only original human studies in the English language were selected (in vitro and animal studies were excluded). Each document was carefully examined by two independent reviewers and information was extracted about enrollment criteria, analytical methods, biological samples, and results. Only studies performed on specimens collected from cord blood or from newborns’ biological fluids were retained for this review.

## 3. Results and Discussion

Our search retrieved 53 references, and 32 of them concerned research on humans. A number of these publications were narrative reviews or commentaries attempting to provide a summary of the biochemical variations associated with IUGR identified using a metabolomic approach. Among these, the most recent and complete was the one performed by Mayneris-Perxachs and Swann [52]. After applying all our inclusion criteria, we finally included 13 original studies in our review, as listed in Table 1. With the exception of Horgan et al., who studied placental villous explants, all the other researchers analyzed newborns’ urine or blood, sampled at birth or in the very first days of life. NMR spectroscopy was used as a platform for metabolomic analysis in five studies, liquid or gas chromatography mass spectrometry in seven, and both in the research published by Bahado-Singh. In some studies, groups were discriminated on the basis of an obstetrical diagnosis of IUGR, in others on weight at birth <10th, 5th, or 3rd centile for postmenstrual age. The study populations also differed in terms of gestational age at birth, ranging from extreme prematurity to full term.

Dessì et al. first compared urinary metabolic profiles of IUGR and AGA preterm infants within 24 and 96 h of birth. Among the discriminant metabolites, they found a significant increase in myoinositol, sarcosine, creatine, and creatinine in the former [53]. Interestingly, an increase in plasma and urine myoinositol levels has been associated with adult glucose intolerance and insulin resistance [54]. A greater excretion of myoinositol—compared with AGA infants—was confirmed not only in IUGR neonates born preterm, but also in those born at term, and in newborns large for gestational age (LGA) [55,56]. This would suggest that a low carbohydrate tolerance is associated with both hypo- and hypernutrition in the uterus. The latest studies have found higher levels of several other metabolites in IUGR newborns, including threonine, citrate, betaine, glycine, urea, glycerol, and uric acid. When Favretto et al. analyzed serum obtained from cord blood at birth, 22 metabolites were able to discriminate between IUGR and AGA newborns, with phenylalanine, tryptophan, and glutamate emerging as the strongest predictors. The authors speculated that phenylalanine and glutamate accumulation in IUGR could be the result of an impaired placental metabolism or transport. Higher levels of tryptophan, a serotonin precursor, could be partially explained by an enhanced brain serotonin synthesis and activity, as seen in animal models of fetal growth restriction [57]. Notably, another study by the same group found a trend (not reaching statistical significance) toward phenylalanine upregulation, and valine, isoleucine, tryptophan, and proline downregulation in selective IUGR monochorionic twins with umbilical artery abnormalities on Doppler ultrasound, when compared with control AGA co-twins [58]. Similarly, Horgan et al. found upregulation of amino acids such as phenylalanine, tryptophan, and methionine in placental villous explants from SGA newborns (compared with AGA babies) that depended on O_2_ tension [59]. Sanz-Cortés et al. used NMR spectroscopy to analyze the metabolomic profile of umbilical cord blood plasma. They confirmed an increase in glutamine and creatine levels in IUGR but found phenylalanine and tyrosine levels lower than normal in cases of IUGR. They attributed this reduction to an altered placental transport combined with a hypercatabolic state in IUGR. The value of their study lies in having compared different clinical subsets of IUGR neonates, namely early- and late-onset IUGR and, among the latter, babies with or without signs of middle cerebral artery vasodilation. The authors suggest that IUGR is a heterogeneous disease, with different metabolic profiles possibly underlying its different clinical presentations [60].

NMR spectroscopy was also used by Moltu et al. to analyze urine samples from 48 premature infants and assess their metabolic status and responses to different nutritional regimens. As a secondary objective, the authors also compared the metabolite profiles of AGA and SGA. They found higher levels of glycine and threonine in the latter, though the difference was not significant at the adjusted significance level (*p* = 0.027 and *p* = 0,033, respectively). This difference in glycine and threonine levels between SGA and AGA children was apparent in the first week of life, but not at later time points (weeks 3, 5, and 7). The authors speculated that increased glycine levels may be caused by a reduced amino acid oxidation or a reduced gluconeogenesis as a strategy to conserve amino acids [61]. In a study by Liu et al., several metabolites (alanine, methionine, ornithine, serine, tyrosine, isovaleryl carnitine, and eicosenoyl carnitine, but not homocysteine) were found in lower concentrations in peripheral blood sampled within 3–7 days of birth in IUGR babies with a birth weight below the 3rd percentile compared to AGA newborns. Interestingly, babies with a birth weight between the 3rd and 5th percentiles showed higher concentrations of the same amino acids, probably due to a compensatory mechanism [62]. Abd El-Wahed matched the metabolic profile obtained from cord blood of 40 SGA and 20 AGA neonates, identifying several metabolites at different concentrations that clearly discriminated between the two groups. Among others, they found elevated levels of acylcarnitine (especially C18-OH and C16-OH), glutamine, leucine, and valine. Glutamine is known to be an important source of cellular energy during fetal life and it has a key role in fetal neurodevelopment. Increased glutamine levels could be explained by a hypercatabolic state associated with IUGR to compensate for the lack of other energy substrates, such as glucose. Acylcarnitines are also important for energy metabolism, as they are involved in fatty acid oxidation. Abnormal plasma acylcarnitine levels have been associated with type II diabetes [63]. Wang et al. recently compared umbilical cord and placental blood collected from 15 selective IUGR twins from monochorionic diamniotic pregnancies with 24 pairs of twins from uncomplicated pregnancies and 14 IUGR singletons. Untargeted metabolomic analyses revealed some metabolites capable of discriminating between the selective IUGR twins and the control twins. Methionine-cysteine, phenylalanine, and tyrosine metabolism were among the metabolic pathways apparently most involved. The added value of this study lies in the comparison drawn within pairs of selective IUGR twins (i.e., between the larger and smaller twin), which led to the identification of metabolites that correlated exclusively with birth weight discrepancy. Another intriguing finding was the higher level of environmental xenobiotics (identified as cyclic siloxanes) associated with selective IUGR [64]. Bahado-Singh et al. were the first to examine the combined use of 1H NMR and LC-MS to compare the biochemical profiles of cord blood serum collected from 40 IUGR patients and 40 controls. The authors provide a detailed description of the strict method used in this analysis. Three variable selection algorithms were tested to identify the most robust panel of potential metabolic biomarkers for detecting IUGR. Creatinine, acetylcarnitine, butyryl carnitine, three lysophosphatidylcholines, and one phosphatidylcholine were the “overlapping” metabolites identified as important by all three selection methods. Using a metabolite set enrichment strategy, the raw data were analyzed to identify the metabolic pathways significantly perturbed in IUGR, which included: beta oxidation of very long fatty acids, oxidation of branched-chain fatty acids, phospholipid biosynthesis, lysine degradation, methionine metabolismthe urea cycle, and fatty acid metabolism. Since most of these metabolic activities involve the liver, the authors suggested that their results might indicate an abnormal fetal liver function in IUGR [65].

It is worth adding some comments on the data analysis methods used in the above-described studies. The analytical platform used for NMR and MS-based metabolomics produces large, complex data sets where redundancy, collinearity, and noise are unavoidable.

The perturbation of a given pathway generates perturbations in the metabolic concentrations of metabolites involved in other pathways because these pathways are not independent, and a single metabolite can produce more than one signal at analytical level. The number of samples is also usually smaller than the number of features being measured, so short and fat data matrices are obtained, and this carries a false discovery risk. Discovering the information hidden in the data is consequently a challenge, and it is essential to use suitable data analysis approaches. To conduct a comprehensive data analysis, the properties of single variables and those related to the cooperative effects of two or more variables should all be investigated. That is why both univariate approaches based on *t*-tests or Mann–Whitney tests with false discovery rate correction (or more complex univariate methods), and multivariate approaches based on Projection to Latent Structures (PLS) regression, Support Vector Machines, or other machine learning tools are necessary. The results of univariate and multivariate data analyses should then be merged and discussed within the same framework. Over-representation pathway analysis can be used to better clarify the role of the features selected using univariate and multivariate methods. Most of the studies considered did not include the use of both approaches, so their data analysis was not exhaustive. When univariate methods were used, most of the studies did not report whether the statistical assumptions were verified. When multivariate approaches were used, model validation (if any) was only done by cross-validation—a simple, rough strategy that is not robust enough when the number of samples is small. Only *n*-fold cross-validation with a single value of *n* was performed in most of the studies, and the model’s behavior under permutation testing or with different values of *n* was not discussed. The lack of an external validation set and a loosely applied internal validation bring into question the reliability of the reported findings. In short, the results presented in most of the studies should only be used for the purpose of generating hypotheses.

In addition, most of the studies did not look into the effects on the metabolome of pharmacological treatments or nutrition. There was also no mention of potential confounding factors in the experimental design. Some of the studies considered babies diagnosed as IUGR during pregnancy, while others relied on SGA definition, namely only on birth weight percentile (probably including some healthy babies who were merely constitutionally small). All these weaknesses limit the feasibility of generalizing the results of the studies considered.

Metabolomics has the extraordinary potential of exploring the whole collection of metabolites present in humans, not only those produced by the humans themselves but also from the commensal communities of microoganisms. Worldwide research projects are attempting to map the human microbiome, giving insight into new species and genes and revealing new roles of the microbiome in human health and disease. Noteworthy, recent molecular findings of bacterial DNA in the in utero environment and meconium challenge the paradigm of the womb as a sterile cavity. It would be of great interest to investigate if and how pregnant mothers’ health influences infants’ microbiota [66]. Interestingly, recent studies suggest alterations in microbial composition not only in overweight and/or insulin-resistant pregnant women, but also in the meconium of infants born to mothers with gestational diabetes compared to controls [67]. Regardless of whether there is some exposure in utero, it is well known that soon after birth, a multitude of microbes colonizes the infant. Type of delivery, early antibiotic exposure, and type of feeding (breast vs. formula milk) seem to impact significantly the gut microbiota with potential lifelong consequences. Moreover, the babies who experience neonatal intensive care, because of prematurity or intrauterine growth restriction, are exposed to many noxae, which may presumably alter their microbiome. For instance, a recent study performed on a murine model shows that exposure to both hyperoxia and antibiotics early in life may alter the intestinal microbiome [68]. An impaired structure and function of the intestine and/or an imbalanced gut microbiota might probably play a role in the mechanisms of IUGR-induced metabolic disease, too. Huang et al. recently found that intestine structure and gut microbiota colonization were impaired in intrauterine restricted piglets when compared to controls, thus altering inflammatory and plasmatic metabolome profiles of the animals [69].

The integration between data from metabolome and microbiome research applied to humans might provide new insights into the relationship between the IUGR condition and metabolic/immune disease later in life.

## 4. Conclusions

Between 5% and 10% of fetuses are unable to achieve their genetically determined potential size as a result of IUGR, and newborns who have experienced IUGR are exposed to short- and long-term health consequences.

Though still preliminary, all the metabolomic studies on this issue have clearly discriminated between IUGR babies and controls. Efforts to pool and interpret the metabolomic data generated so far proved very complicated and challenging, however. This is partly due to: nonhomogeneous study populations (preterm and term infants), different analytical approaches (NMR or MS), small sample sizes, and inconsistencies in the reported results. Most of the studies lack a well-defined experimental design, which compels us to be cautious about generalizing the findings.

Despite these limitations, the metabolic perturbations found in IUGR neonates to date allow us to hypothesize an “early” pattern of glucose intolerance, insulin resistance, catabolite accumulation, disrupted amino acid metabolism, and abnormal fetal liver function (Figure 1).

Future research on metabolomics in IUGR will hopefully rely on better-defined bioinformatic modeling strategies and methods. In addition, narrower inclusion criteria, able to discriminate between newborns who are IUGR and babies who are only constitutionally small, should be ideally applied in the future.

Metabolomics could help us to detect otherwise unknown nutritional deficiencies in IUGR preterm infants lacking the third-trimester accrual of several macro- (proteins, lipids) and micronutrients (calcium, iron, phosphate). It could represent a valid tool for discriminating between “good growth” and “excessive growth”, the latter orienting the metabolism toward an atherogenic or obesogenic profile, especially in newborns who have experienced IUGR. Obtaining such information with a metabolomic approach could help neonatologists further improve the quality and appropriateness of newborn infants’ growth.

## Figures and Tables

**Table 1:** Postnatal metabolomic studies on fetal growth restriction.

Study	Subjects	Sample	Method	Results
Dessì (2011) [53]	26 IUGR vs. 30 AGA (preterm)	Urine within 24 h at 96 h	^1^H-NMR	⇑ myoinositol, sarcosine, creatine, creatinine
Horgan (2010) [59]	9 SGA (BW < 5th %ile) vs. 8 AGA (term)	Placental villous explants	LC–MS	Difference in metabolite levels between the two groups, depending on O_2_ tension exposure
Favretto (2012) [57]	22 IUGR vs. 22 AGA (GA: 32–41 weeks)	Cord vein blood	LC-MS	⇑ phenylalanine, tryptophan and glutamate, methionine, proline, valine, isoleucine, dopamine, histidine, uric acid, caffeine, 5-methyl-2-undecenoic acid, oleic acid, 1-hydroxyvitamin D3 3-D- glucopyranoside, L-thyronine, hexadecanedioic acid
Cosmi (2013) [58]	4 selective IUGR MCDA twins vs. 4 AGA MCDA twins (GA: 28–36 weeks)	Cord vein blood	LC-MS	⇑ phenylalanine, sphingosine, glycerophosphocholine
				⇓ valine, tryptophan, isoleucine, proline, choline (not statistically significant)
Sanz-Cortés (2013) [60]	20 early IUGR (GA: 31.7 ± 2.2 weeks) vs. 23 matched AGA	Umbilical vein blood	^1^H-NMR	⇑ VLDL, unsaturated lipids, acetone, glutamine, creatine
				⇓ glucose, phenylalanine, tyrosine, choline
	56 late IUGR (mean GA: 38.3 ± 1.9 weeks) vs. 55 matched AGA	Umbilical vein blood	^1^H-NMR	⇑ VLDL, unsaturated lipids
				⇓ phenylalanine, glutamine tyrosine, choline, valine, leucine
Barberini (2014) [54]	11 IUGR (+ 12 LGA) vs. 10 AGA (mean GA: 37 weeks)	Urine ● within 12 h	GC-MS	⇑ inositol
				≠ urea, glycerol, glucose, citric acid, uric acid
Dessì (2014) [55]	12 IUGR +12 LGA vs 17 AGA (GA: 34–41 weeks)	Urine ● within 8 h	^1^H-NMR	⇑ myoinositol, creatinine, creatine, citrate, betaine, glycine
				⇓ urea, aromatic compounds and branched chain amino acids
Marincola (2015) [56]	8 IUGR vs. 8 AGA (mean GA: 36.9 vs. 37.5 weeks)	Urine within 8 h at 4 days at 7 days	^1^H-NMR	⇑ myoinositol, citrate, glycine,
				⇓ succinate, betaine, creatinine
Moltu (2014) [61]	16 SGA vs. 32 AGA (mean GA: 29.9 vs. 27.5 weeks)	Urine ● within 1 week	^1^H-NMR	⇑ glycine, threonine (not significant when adjusted for gestational age at birth)
Liu (2016) [62]	25 SGA (BW < 3rd %ile) vs. 60 controls (mean GA: 36.8 vs. 35.9 weeks)	Blood spot ● between 3 and 7 days	Targeted LC-MS	⇑ homocysteine
				⇓ alanine, methionine, ornithine, serine, tyrosine
Abd El-Wahed (2017) [63]	40 SGA vs. 20 AGA (mean GA: 34 ± 2.4 vs 35 ± 1.4 weeks)	Umbilical cord blood spot	Targeted LC-MS	⇑ several acylcarnitines including C18-OH, C16-OH, alanine, arginine, aspartate, citrulline, glutamine, isoleucine, leucine, ornithine, phenylalanine, tyrosine, valine
				⇓ histidine, methionine
Wang (2018) [64]	15 pairs of selective IUGR MCDA twins vs. 24 pairs of uncomplicated MCDA twins (mean GA: 35 vs. 36.5 weeks)	Umbilical cord blood	GC-MS	⇑ methionine, phenylalanine, 4-hydroxyphenylacetic acid, 2-aminobutyric acid, decamethylcyclopentasiloxane, tyrosine, isoleucine, eicosapentaenoic acid
				⇓ adrenic acid
Bahado-Singh (2019) [65]	40 IUGR vs. 40 controls (mean GA not known)	Umbilical cord blood serum	LC-MS + ^1^H-NMR	≠ creatinine, acetyl carnitine, butyryl carnitine, lysophosphatidylcholines (C16.1, C20.3, and C28.1) and phosphatidylcholine C24:0

IUGR: intrauterine growth restriction; AGA: adequate for gestational age; ^1^H-NMR: proton nuclear magnetic resonance spectroscopy; SGA: small for gestational age (BW < 10th %ile unless specified); LC-MS: liquid chromatography mass spectrometry; GA: gestational age; MCDA: monochorionic diamniotic; GC-MS: gas chromatography mass spectrometry; BW: birth weight.
